# ‘Mind the gap’ - mapping services for young people with ADHD transitioning from child to adult mental health services

**DOI:** 10.1186/1471-244X-13-186

**Published:** 2013-07-10

**Authors:** Charlotte L Hall, Karen Newell, John Taylor, Kapil Sayal, Katie D Swift, Chris Hollis

**Affiliations:** 1 CLAHRC-NDL, University of Nottingham, Nottingham, UK; 2 Developmental Psychiatry, University of Nottingham, Queen’s Medical Centre, Nottingham, UK; 3 University of Manchester, Manchester, UK; 4 B07 Institute of Mental Health, University of Nottingham, Triumph Road, Nottingham NG7 2TU, UK

**Keywords:** Transition, Service mapping, ADHD, Adults

## Abstract

**Background:**

Once considered to be a disorder restricted to childhood, Attention Deficit/Hyperactivity Disorder (ADHD) is now recognised to persist into adult life. However, service provision for adults with ADHD is limited. Additionally, there is little guidance or research on how best to transition young people with ADHD from child to adult services.

**Method:**

We report the findings of a survey of 96 healthcare professionals working in children’s (Child and Adolescent Mental Health Services and Community Paediatrics) and adult services across five NHS Trusts within the East Midlands region of England to gain a better understanding of the current provision of services for young people with ADHD transitioning into adult mental health services.

**Results:**

Our findings indicate a lack of structured guidelines on transitioning and little communication between child and adult services. Child and adult services had differing opinions on what they felt adult services should provide for ADHD cases. Adult services reported feeling ill-prepared to deal with ADHD patients, with clinicians in these services citing a lack of specific knowledge of ADHD and a paucity of resources to deal with such cases.

**Conclusions:**

We discuss suggestions for further research, including the need to map the national provision of services for adults with ADHD, and provide recommendations for commissioned adult ADHD services. We specifically advocate an increase in ADHD-specific training for clinicians in adult services, the development of specialist adult ADHD clinics and greater involvement of Primary Care to support the work of generic adult mental health services in adult ADHD management.

## Background

Attention Deficit/Hyperactivity Disorder (ADHD) has been described as a ‘chronic illness’, categorised by core symptoms of inattentiveness, impulsiveness and hyperactivity, which affects up to 5% of children in the UK (National Institute for Clinical Excellence, [1]). There is now a recognition that impairments associated with ADHD persist into adulthood in around two thirds of cases [2], with approximately 2.5% of adults meeting diagnostic criteria for ADHD [3]. ADHD in adulthood is associated with an increased risk of car accidents, higher rates of divorce and substance misuse, and more frequent job changes [2,4,5]. Given the impact of ADHD in adult life, it is vital that adolescents with ADHD are able to access continuing support from mental health services as they transition into adulthood. Despite this recognised need, there is little published research on the transition of those with ADHD from child and adolescent mental health services (CAMHS) to adult mental health services (AMHS), and what adult services should provide.

UK Government guidelines [6] have begun to make recommendations for improving the transition process from child to adult health services [7]. However, these guidelines do not specifically address the issues involved in the transition from child to adult *mental health* services. Recent NICE [1] guidelines have recognised the importance of ADHD in adults and identified three groups with ADHD service requirements; adults who are currently treated for ADHD, adults diagnosed with ADHD in childhood but currently untreated (often lost in transition from CAMHS) and adults with symptoms of ADHD but who have never received a clinical diagnosis. NICE recommend that services for adults should include drug monitoring, psychological services and diagnostic services. Furthermore, NICE have suggested that important factors for the development of transition for ADHD cases include timing of transition, transition planning and joint working between CAMHS and AMHS.

One of the most thorough investigations of the transition process within mental health has come from the TRACK project which investigated the transition from CAMHS to AMHS across a range of mental health diagnoses. As part of this, Singh et al. [8] surveyed 42 CAMHS teams in Greater London who reported transitioning young people to adult services. Their findings showed that in 2005 there were 13 different transition protocols operating in Greater London. All policies were underpinned by similar principles including ensuring consistency in the service, providing a seamless transition, information sharing between services, joint working, clarity about clinicians’ roles and user and carer involvement in decision making. However, they differed on key points such as age of transition, flexibility of boundaries, joint working between CAMHS and AMHS and whether protocols were shared at Trust or local level. Specific omissions in all protocols included how to prepare young people for transition and how to ensure continuity of care for individuals who were not accepted into adult services. The transition pathway from CAMHS to AMHS varied widely, and whilst young people with severe mental health disorders were more likely to transfer to AMHS, those with neurodevelopmental disorders (including ADHD) were more likely not to meet acceptance criteria for AMHS. These findings were confirmed by a recent longitudinal investigation which tracked a cohort of young people transitioning from CAMHS to AMHS across six mental health Trusts, three of which were in Greater London and three in the West Midlands [9]. Singh et al. [9] found that young people who had a severe mental illness or were taking medication were more likely to transition to AMHS than those with neurodevelopmental disorders such as ADHD and autism spectrum disorder (ASD). Of the 90/150 young people who did transition to adult services, less than 5% received ‘optimal’ transition (consisting of joint working between CAMHS and AMHS, transition planning and information transfer across teams). In support of this, a recent study [10] audited the transition of ADHD patients from CAMHS to AMHS and found that although 104 young people were eligible for transition, 73% of these were discharged or lost during follow-up. The findings clearly highlight that young people with neurodevelopmental disorders (including ADHD) are at high risk of failing to transition successfully to adult services.

There is a paucity of research investigating the current provision of adult services for young people with ADHD; however a couple of studies have begun to examine this. Marcer et al. [11] surveyed 78 consultant Community Paediatricians regarding their experiences of transferring patients to adult services. Of the 68 respondents who saw young people with ADHD, 40% felt their patients would need continuing treatment into adulthood and 90% felt there was a need for a specialist adult ADHD clinic. Although this research emphasises the importance of specialist and continuing care into adulthood, it does not detail the provision of adult services for ADHD cases. Another study assessed 38 AMHS clinicians’ attitudes and practices towards ADHD in adults [12]. This survey showed that 50% (19/38) of clinicians working in AMHS felt confident enough to diagnose ADHD despite the fact that 90% had no direct experience of diagnosing or treating adults with ADHD. The majority of respondents identified a service gap when transitioning from CAMHS to AMHS and felt that they needed further training to enable them to aid their ability to diagnose and treat ADHD patients. Crucially, this research highlights that AMHS clinicians perceive that they lack the training and skills to assess and manage adults with ADHD. Given that adults with ADHD can experience significant impairment [13], it is vital that adult mental health services are equipped with the necessary training and understanding to manage such cases. In order to improve the transition pathway and plan future service developments for adults with ADHD, it is imperative that we gain a better understanding of all aspects of this process. As part of this, we should understand how transition is experienced from the perspective of clinicians working in CAMHS, Community Paediatrics and AMHS, and the current provision in adult mental health services. To date, no research has mapped the provision of current care for adults with ADHD and the efficacy and acceptability of current treatment practices for this vulnerable patient group is unknown.

By defining current services for adults in three East Midlands’ counties in the UK, we will provide a crucial starting point to identify gaps in care and document what clinicians require in order to improve service provision for adults with ADHD. Eliciting the views clinicians have about the transition process, the diagnosis of ADHD and what form ADHD services for adults should take, offers a unique and much needed understanding about this under-documented and often turbulent process.

This study forms part of the wider TRAMS (Transition to Adult Mental Health Services) project which is investigating the transition of young people with ADHD into adult services. The research is funded by NIHR (National Institute of Health Research) CLAHRC-NDL (Collaborations for Leadership in Applied Health Research and Care – Nottinghamshire, Derbyshire and Lincolnshire) which aims to understand and facilitate the complex processes involved in translating research into routine clinical practice.

## Method

### Procedure

Healthcare Professionals (HCPs) working within NHS Trusts in the East Midlands region of England (Nottinghamshire, Derbyshire and Lincolnshire) covered by the NIHR CLAHRC-NDL were invited via an email to participate in an on-line survey hosted by http://www.surveymonkey.com. This incorporated five NHS Trusts (Nottinghamshire Healthcare NHS Trust (NHT), Nottingham University Hospitals NHS Trust (NUH), Sherwood Forest Hospitals NHS Foundation Trust (SFHT), Derbyshire Healthcare NHS Foundation Trust (DHFT) and Lincolnshire Partnership NHS Foundation Trust (LPFT). The three regions have a combined population of approximately 2 million (Nottinghamshire = ~1.1million, South Derbyshire = ~ 342,000, Lincolnshire = ~715,000; http://www.ons.gov.uk). HCPs were informed that the survey aimed to explore issues for young people with ADHD who needed to transfer from children’s services to AMHS. Questions centered on the characteristics of the service they worked in (child or adult), transitioning, adult services for ADHD and attendance rates. Data were collected between August and December 2012 and downloaded from the website at the end of the study and descriptive statistics were used.

### Respondents

The survey was sent out to a total of 498 HCPs of which 96 staff responded (19%), although not all fully completed the survey. As data on the provision of care for young people with ADHD are scarce, the survey was distributed to all adult/child services wherever possible to maximise inclusivity and to ensure that our mapping was comprehensive. However, the survey was not applicable to HCPs who did not have direct responsibility for the care of ADHD patients, which may explain our relatively low response rate. Furthermore, 13 respondents (14%) reported they were replying on behalf of a team.

The sample comprised 33 Psychiatrists, 28 Nurses, 22 Paediatricians, 4 Psychologists, 4 Social Workers, 2 Psychotherapists, 2 Managers, and 1 Occupational Therapist. Of the sample, 26% (22/90) were from CAMHS, 24% (22/90) from Community Paediatrics, 42% (38/90) from AMHS and 4% (4/90) from Learning Disability Services. Ethical approval for the study was granted by the local Research Ethics Committee and Research and Development Departments of NHT, SFHT, NUH, LPFT and DHFT. Approval from the Chief Executive of each Trust was also obtained.

## Results

### The transition process

Respondents from both child and adult services typically reported a lack of clarity about the transition process. All (24) respondents across adult services (in five NHS Trusts) reported having no transition policy. In contrast, 55% (20/36) of respondents from child services (CAMHS and Community Paediatrics) reported having a written transition policy, indicating a greater awareness of transition protocols among those working in children’s services. Most respondents (89%, 31/35) from child services reported having no dedicated transition staff although 72% (23/32) felt that transition staff would be beneficial.

Responses from child and adult services were comparable when asked about joint working between CAMHS and AMHS. From child services, 66% (23/35) reported no periods of joint working with AMHS, from adult services 59% (13/22) reported no joint working with CAMHS.

There was a large variation in responses about boundaries for transition/acceptance of ADHD cases. There was a tendency for both services to report age alone as the key criteria for transition/acceptance (child services = 53%, 20/38; adult services = 26%, 6/23). However, other criteria for transition from child services included a combination of age and severity of condition (18%, 7/38) and availability of a service to refer on to (8%, 3/38). Factors including the presence of a learning disability, commissioning policy, patients’ need for on-going support, presence of a co-morbid disorder, education status, assessment based on the type of problem, assessment on a case-by-case basis and always referring to adult services were each cited as criteria by 2.6%, (1/38) respectively.

Other criteria for acceptance by adult services included accepting any referral from CAMHS or a GP (15%, 4/23) or a combination of age and the presence of co-morbid Autism Spectrum Disorder (ASD; 9%, 2/23), co-morbid psychosis (9%, 2/23), or a learning difficulty (9%, 2/23). Four per-cent (1/23) also reported the following: severity of condition, predisposition to violent offending, substance misuse and learning disability regardless of age. Despite the lack of agreement on specific boundaries for transition/acceptance, 68% (26/38) of respondents from child services and 74% (17/23) of respondents from AMHS felt their boundaries were appropriate.

Table 1 shows the perceptions of child service HCPs regarding which ADHD cases are most likely to be accepted into adult services. Although there was some variation in responses, respondents reported that patients who had co-morbid mental health problems, a learning disability or required medication were usually seen by adult services. Only just over half of HCPs in child services (*n* =19, 53%) felt that young people prescribed medication for ADHD would meet acceptance criteria for AMHS. Given that ADHD medications are generally not licensed in adults and are typically not covered by shared care protocols (SCPs) with Primary Care, reluctance of AMHS to accept responsibility for on-going prescribing and monitoring is likely to result in young people having to stop treatment.

**Table 1:** Child service respondents’ perceptions of the characteristics of young people with ADHD who are accepted by adult services

**Characteristic**	Child services respondents (%)*
	***N *= 36**
Co-morbid mental health problem	81% (29)
Learning disability	56% (20)
Medication being prescribed	53% (19)
Other co-morbidities (e.g., conduct)	33% (12)
Special educational needs	25% (9)
Forensic history	14% (5)
Looked after child status	14% (5)
Co-morbid physical health problem	11% (4)
Other	14% (5)

*Note*.*Responses represent the percentage of respondents who reported that these cases would be accepted.

The most commonly reported reason why adult service respondents felt that transition for ADHD cases can be unsuccessful was because the young person fails to meet the service threshold for acceptance (63%, 19/30). The other major reason cited was related to difficulties young people experience in engaging with AMHS. Twenty per-cent (6/30) felt it was because the young person does not attend their appointments and 17% (5/30) felt it was because the young person did not wish to be seen by adult services.

### Provision of adult services for ADHD – AMHS perspective

To further understand services for adults with ADHD, we elicited the views of how HCPs working in adult services viewed the provision of this care. There was an overwhelming agreement (82%, 18/22) on the lack of provision of services for adults with ADHD. Further examination of responses revealed that the majority of respondents felt that they lacked training or knowledge specific to the condition (95%, 21/22) and that they did not have adequate resources for seeing patients with ADHD (86%, 19/22). There was support for the involvement of GPs in the management of adults with ADHD, with 86% (19/22) of respondents agreeing this should be the case. Seventy-seven per-cent (17/22) of all respondents across the five Trusts surveyed felt their service would be more likely to accept patients with ADHD if consultation with a specialist ADHD clinic was available.

Opinion was divided as to whether differences in working styles across CAMHS, Community Paediatrics and AMHS created difficulties caused by unrealistic expectations of the family involvement in adult services. Fifty-nine per-cent (13/22) reported experiencing difficulties with engaging families due to the transition from the more systems-based working used in CAMHS to the more individual-focused approach used in AMHS, whereby the family is less involved in the intervention.

### Provision of adult services for ADHD – comparing CAMHS & AMHS perspective

Across child and adult services there were differing views about what AMHS involvement in adult ADHD should be. The results are displayed in Table 2, and highlight a lack of consensus amongst adult and child services health professionals about what should be provided. All child service respondents (100%, 24/24) felt that AMHS should provide services for adults with ADHD; however, agreement was less for adult service respondents (68%, 15/22).

**Table 2:** Perceptions of what adult and child services feel AMHS involvement should be in adults with ADHD

**AMHS role in ADHD management**	**Adult services - *N *= 22**	**Child services - *N *= 25**
Monitoring and prescribing for all cases transitioning from CAMHS	45% (10)	84% (21)
Monitoring and prescribing only for patients with complex and comorbid ‘adult’ disorders	50% (11)	36% (9)
New ADHD diagnostic assessment from Primary Care	64% (14)	78% (20)
Assessment and management of ‘old’ ADHD cases (diagnosed in CAMHS) returning as adults for treatment	75% (17)	89% (22)
Assessment and management of existing AMHS patients for possible ADHD	73% (16)	93% (23)

There was a notable divide in the confidence of child and adult services in dealing with ADHD. Whereas 96% (24/25) of respondents from child services felt they possessed the necessary skills to assess and manage people with ADHD, only 54% (12/22) of HCPs in AMHS felt this to be the case. Seventy-seven per-cent (19/22) of adult service respondents felt they would like more training in this area.

There was a general consensus supporting the provision of a specialist adult ADHD clinic, with 88% (22/25) and 73% (16/22) of child and adult service respondents endorsing this respectively. However, exactly what form this clinic should take varied across child and adult services. Respondents from child services tended to feel that this service should be a secondary level of care for all adult cases (65%, 15/23), whereas respondents from adult services were more likely to suggest the service should include a tertiary level specialist adult ADHD clinic which provides management and advice to AMHS (69%, 11/16).

Table 3 shows that most respondents felt ADHD to be a valid diagnosis in adults, although HCPs in adult services were less confident about the effectiveness of treatments for ADHD.

**Table 3:** Adult and child service respondents’ opinions on the validity of an ADHD diagnosis and ADHD treatments for adults*

	**ADHD diagnosis**		**ADHD treatment**	
	**Valid**	**Not valid**	**Effective**	**Not effective**
Adult services *N* = *22*	73% (16)	14% (3)	41% (22)	14% (3)
Child services *N* = 25	84% (21)	0% (0)	60% (15)	4% (1)

*Respondents were also given the option to select a neutral ‘neither agree nor disagree’ option.

The majority of respondents from both services reported a perceived need for an ADHD transition support worker (child services = 88%, 22/25; adult services = 75%, 13/18).

### Attendance in child and adult services

There was a marked difference in reported attendance of young people with ADHD between child and adult services. All (25) respondents from child services reported that young people with ADHD regularly attended their appointments, whereas only 54% (12/22) of respondents from adult services reported regularly attended appointments.

There was limited reported use of appointment reminders being utilised across the two services. For child services, 43% (9/21) reported using a reminder service; this figure was slightly less for adult services with 32% (8/25) reporting sending out reminders. All respondents who reported using reminders specified text messages or telephoning as their method.

## Discussion

This study sought to understand the provision of services for young people with ADHD transitioning into adult services by eliciting the opinions of healthcare professionals working with ADHD cases and spanning children’s services (CAMHS and Community Paediatrics) and adult mental health services in five NHS Trusts across the East Midlands region of England, serving a population of approximately 2 million people. In mapping the perceptions of transition and the characteristics of existing adult services for ADHD within these NHS Trusts, we have highlighted a lack of clarity surrounding the transition process. This is exacerbated by inadequate resources, limited communication between child and adult services, and adult services often feeling ill-prepared to deal with ADHD patients.

The findings support and extend existing studies investigating transition from CAMHS to AMHS. Similar to the findings of Singh et al. [8], there was limited evidence of any written transition protocols within child services. However, our findings indicate that the lack of transition guidelines was a particular problem for adult services, with no respondents indicating having, or being aware of, such a protocol. Despite government and NICE guidelines highlighting the importance of transition from child to adult health services, there is a clear need for a more structured approach to transition protocols that are adopted by HCPs in both children’s and adult services. In response to this need, Young et al. [14] provide a more extensive set of guidelines for commissioners and providers of healthcare services on how to manage the transition process for young people with ADHD.

The lack of clear NICE guidelines on transitioning may explain the variation in responses regarding the transition boundaries and criteria from individual respondents working in both child and adult services. Consistent with earlier TRACK studies [8,9], respondents perceived patients with a co-morbidity or those taking medication as being more likely to be accepted by adult services. However, reported criteria and their boundaries for transitioning were heterogeneous and lacked clear consensus. The fact that NICE guidelines recommend that transition is complete by the age of 18 years could explain why age was a frequently reported criterion for transition. However, the numerous other criteria cited in the survey indicate a lack of consensus amongst HCPs regarding agreed and consistently applied criteria for transition to adult services. This lack of consensus may underpin previously reported anxieties that HCPs working in child services have regarding transferring their patients to adult services (e.g. transition of a young person has to be negotiated on a ‘case by case’ basis) and the difficulties associated with getting young people accepted by AMHS [14]. It is possible that this reflects lack of agreement on what is ‘best procedure’ in these cases and the need for local implementation of NICE guidelines on the principles of transition. It should be noted that NICE guidelines are not mandatory and may not be adhered to due to a lack of resources, or knowledge in relation to ADHD in adults and how best to transfer these cases [15]. The findings also highlight the issue of joint working and formal meetings between CAMHS and AMHS, a recommendation made by the NICE ADHD guideline [1]. In support of the TRACK studies [8,9] there was a notable lack of joint-working between the two services.

Another notable discrepancy with NICE [1] recommendations related to HCPs perceptions of what AMHS involvement with ADHD cases should be. Although the majority of respondents from child services agreed with the NICE guidelines that AMHS should assess and manage cases diagnosed in CAMHS, under half of the respondents working in adult services felt this should be the case. Instead, HCPs working in adult services appeared to support a more selective role for generic AMHS in ADHD management. Specifically, respondents from adult services indicated that they would like Primary Care to be responsible for transition cases that only require routine monitoring and for a specialist adult ADHD clinic to see ‘new’ adult cases that require diagnostic assessment. However, they saw a greater role for adult services in managing ADHD within the existing AMHS caseload, and also in young people returning to services who were previously treated as children. Although these findings suggest that AMHS see a limited role for themselves in diagnosing and treating ADHD cases, it is possible this may reflect a lack of confidence in dealing with ADHD which could be improved by increased training. Young et al. [14] discuss different models of healthcare provision for ADHD transition and suggest that shared care arrangements between Primary and Secondary Care services merit further investigation. They suggest that GPs could be responsible for transitioning patients who are responding positively to medication, but less stable cases should be referred to general AMHS or if necessary, specialist services. If AMHS do not take responsibility for diagnosing ADHD (as findings from our survey suggest) it is likely that a diagnosis of ADHD could be over-looked (false negatives) or made in error (false positives). With further training, AMHS clinicians may feel more able to make this diagnosis accurately.

The clear difference in attendance of young people at child and adult clinics may be explained by family discontentment arising from cultural and attitudinal differences between child and adult services. Whereas child services typically adopt a more developmental and family systems approach to intervention, AMHS take a more medical and individual-focused perspective [16]. As a result, adult services are less likely to include parents in appointment letters and in the intervention process [9], thereby placing greater responsibility of treatment adherence and appointment attendance on the young person. These issues were reflected in our survey, whereby some respondents felt that differences in working styles between CAMHS and AMHS created difficulties for families and young people at the point of transition.

The findings showed that respondents from adult services felt that they lacked training and knowledge to assess and manage ADHD patients. However, it is interesting to note that HCPs in AMHS did not challenge the validity of ADHD as a diagnosis in adults, although they were less certain about the benefits of treatment. This suggests that more information and training on treatments would be helpful and create a more positive approach to treatment and supports earlier work identifying the need for further ADHD training for adult HCPs [12]. Although, in comparison to childhood ADHD, there is little research investigating the effects of treatments for ADHD in adults, the available evidence suggests that Methylphenidate is an effective drug for treating ADHD in adults [17] and that therapeutic intervention (such as cognitive therapy) used in conjunction with medication is also effective [18,19]. However, if left untreated, ADHD is associated with significantly higher likelihood of difficulties in basic functioning (self-care, mobility and cognition), instrumental functioning (social-role and productiveness) and poorer outcome than for individuals who are treated for ADHD [20,21]. Clearly, there is evidence for the effectiveness of interventions for ADHD in adulthood and given that ADHD persists into adulthood in up to two-thirds of cases, there is a strong need for services for adult ADHD. As well as cases potentially transitioning from CAMHS, many undiagnosed cases of ADHD first present for help during adulthood. Adult ADHD is associated with considerable comorbidity and functional impairment; both pharmacological and psychological interventions improve outcomes [22].

Given that ADHD has typically been seen as a ‘childhood disorder’ [5], this could be a reason for adult clinicians feeling under-equipped to deal with such cases. It is possible that clinicians’ lack of experience and confidence in dealing with ADHD patients alongside their beliefs about the effectiveness of treatments may also be leading to patient discontentment with adult services, suggested by the lower attendance figures. Furthermore, if HCPs in AMHS are unsure about treatment effectiveness they are less likely to engage patients in treatment in the first place.

One cost-effective solution might be to provide AMHS clinicians with further training and jointly develop clear protocols for diagnosing and treating ADHD in adults. This increased training would hopefully increase clinicians’ confidence in dealing with ADHD treatments and lead to better provision of services for adults with this diagnosis. Another potential solution to improve care for young people/adults with ADHD is to develop more specialist adult ADHD clinics; a suggestion supported by the majority of HCPs from both child and adult services. This would ensure ADHD patients have access to specialist on-going care even if they do not have a more severe mental illness typical of adult mental health patients [23]. Our findings also highlight the possibility of increasing the involvement of Primary Care to support these specialist clinics. For example, involvement of Primary Care could be facilitated by shared care protocols (SCPs) and Primary Care could be responsible for cases that are without major psychiatric co-morbidities and only require routine monitoring for ADHD medication. This would allow specialist clinics, which are likely to be small in size, to focus on the diagnosis of new cases and providing more specialist support to AMHS for more complex cases with psychiatric co-morbidities.

Our findings indicated a difference in HCPs’ opinions as to what form a specialist clinic should take. Whereas respondents from child services tended to feel the clinic should be a secondary level of care for all adult cases, respondents from adult services felt it should be a tertiary level of care. Based on our findings, we suggest that the specialist clinic could support AMHS HCPs in assessment and management of their cases by providing expert consultation, advice and training. Verity and Coates [24] provide a short report on a specialist transitional ADHD clinic running in South Yorkshire. However, this clinic appears to focus on providing continuing medication for patients during the transition period and does not provide more consistent regular support or any therapy-based intervention. Unfortunately, to date there is no published information on the success of this project. Future research should further investigate exactly what form a specialist clinic should take to fulfil the needs of young people with ADHD transitioning in to adult services.

### Strengths and limitations of study

To our knowledge, this is the first survey that gathers and compares the opinions of transition from both adult and child services, with previous studies typically focusing on the opinions of child services [8,9,11]. The survey also incorporates views of Paediatricians who are responsible in many areas of the UK. for delivering a large proportion of ADHD services for children and young people. A further strength is that the survey covers five NHS Trusts serving a population of almost 2 million people in England. In doing so we provide novel information on the provision of adult services; a fundamental step to aid the development of future commissioned services to improve the provision of care for adults with ADHD. However, the findings need to be considered in light of our relatively low response rate and focus on one geographical region of the UK. Although the response rate was smaller than that in comparable studies [8,9], this is likely due to our decision to distribute the survey to all healthcare professionals working in adult and child services even though we only wanted to utilise responses from individuals who had contact with ADHD cases. It had been felt that this service-wide distribution approach would best encapsulate views from all professionals working with ADHD that may otherwise be overlooked. Caution should be taken when generalising the findings of this survey to other Trusts located in different geographical regions and clearly the next step is to map the provision of ADHD services both nationally and internationally. Despite this, our findings are consistent with the TRACK study [8,9] conducted in Greater London and the West Midlands, giving further support to their reliability.

## Conclusions

The findings from this survey provide a valuable and up until now under-researched insight into the provision of services for young people with ADHD transitioning into adult services. Although the relatively small sample size means that the generalizability of the findings should be exercised with caution, they clearly demonstrate the need for clear guidelines to be adopted for transitioning ADHD cases. Additionally, HCPs in adult services require more training to effectively manage such cases, which may also increase clinician awareness of ADHD in adults. It is evident that there needs to be more joint-working and information sharing between child and adult services, including preparing the young person for any change in working style that is utilised by AMHS. It is not only generic AMHS that need to rise to the challenge of managing adult ADHD. The role of Primary Care and specialist adult ADHD clinics working together in an integrated fashion with AMHS is likely to be essential if services are to address the range of needs of adults with ADHD. Such changes require local commissioning of adult ADHD services supported by cultural and attitudinal shifts in perspective by acknowledging that ADHD is a lifespan neurodevelopmental disorder with clinical and care needs extending into adult life.

### Ethical & R & D approval

Ethical approval for the study was granted by the NRES Committee East Midlands – Derby (REC reference 11/EM/0027) and R&D approval was obtained from the five NHS Trusts (Nottinghamshire Healthcare NHS Trust (NHT), Nottingham University Hospitals NHS Trust, Sherwood Forest Hospitals NHS Foundation Trust, Derbyshire Healthcare Foundation Trust (DHFT) and Lincolnshire Partnership NHS Foundation Trust (LPFT).

## Competing interests

The authors declare that they have no competing interests.

## Authors’ contributions

KDS designed the survey. CLH and KN oversaw data collection and analysed the survey results. All authors contributed to the interpretation of the data and the study write-up. CLH drafted the manuscript and KN, JT, KDS, KS and CH revised it critically for important intellectual content. All authors read and approved the final manuscript.

## Pre-publication history

The pre-publication history for this paper can be accessed here:

http://www.biomedcentral.com/1471-244X/13/186/prepub
